# How doctors actually (do not) involve families in decisions to continue or discontinue life-sustaining treatment in neonatal, pediatric, and adult intensive care: A qualitative study

**DOI:** 10.1177/02692163211028079

**Published:** 2021-06-28

**Authors:** A. (Aranka) Akkermans, J.M.W.J. (Joyce) Lamerichs, M.J. (Marcus) Schultz, T.G.V. (Thomas) Cherpanath, J.B.M. (Job) van Woensel, M. (Marc) van Heerde, A.H.L.C. (Anton) van Kaam, M.D. (Moniek) van de Loo, A.M. (Anne) Stiggelbout, E.M.A. (Ellen) Smets, M.A. (Mirjam) de Vos

**Affiliations:** 1 Department of Medical Psychology, Amsterdam UMC, University of Amsterdam, Amsterdam, The Netherlands; 2 Faculty of Humanities, Department of Language, Literature and Communication, VU Amsterdam, Amsterdam, The Netherlands; 3 Department of Intensive Care Medicine, Amsterdam UMC, University of Amsterdam, Amsterdam, The Netherlands; 4 Mahidol-Oxford Tropical Medicine Research Unit (MORU), Mahidol University, Bangkok, Thailand; 5 Nuffield Department of Medicine, University of Oxford, Oxford, UK; 6 Department of Pediatric Intensive Care, Emma Children’s Hospital, Amsterdam UMC, University of Amsterdam, Amsterdam, The Netherlands; 7 Department of Neonatology, Emma Children’s Hospital, Amsterdam UMC, University of Amsterdam, Amsterdam, The Netherlands; 8 Medical Decision Making, Department of Biomedical Data Science, Leiden University Medical Center, Leiden, the Netherlands; 9 Department of Pediatrics, Emma Children’s Hospital, Amsterdam UMC, University of Amsterdam, Amsterdam, The Netherlands

**Keywords:** Qualitative research, critical care, decision making, communication, family, palliative care

## Abstract

**Background:**

Intensive care doctors have to find the right balance between sharing crucial decisions with families of patients on the one hand and not overburdening them on the other hand. This requires a tailored approach instead of a model based approach.

**Aim:**

To explore *how* doctors involve families in the decision-making process regarding life-sustaining treatment on the neonatal, pediatric, and adult intensive care.

**Design:**

Exploratory inductive thematic analysis of 101 audio-recorded conversations.

**Setting/participants:**

One hundred four family members (61% female, 39% male) and 71 doctors (60% female, 40% male) of 36 patients (53% female, 47% male) from the neonatal, pediatric, and adult intensive care of a large university medical center participated.

**Results:**

We identified eight relevant and distinct communicative behaviors. Doctors’ sequential communicative behaviors either reflected consistent approaches—a shared approach or a physician-driven approach—or reflected vacillating between both approaches. Doctors more often displayed a physician-driven or a vacillating approach than a shared approach, especially in the adult intensive care. Doctors did not verify whether their chosen approach matched the families’ decision-making preferences.

**Conclusions:**

Even though tailoring doctors’ communication to families’ preferences is advocated, it does not seem to be integrated into actual practice. To allow for true tailoring, doctors’ awareness regarding the impact of their communicative behaviors is key. Educational initiatives should focus especially on improving doctors’ skills in tactfully exploring families’ decision-making preferences and in mutually sharing knowledge, values, and treatment preferences.

**What is already known about the topic?**

- In intensive care units, decisions about the continuation or discontinuation of life-sustaining treatment often concern decisions in the “gray zone.”
- Decisions in intensive care units often involve family members as surrogate decision-makers.
- Families vary in their preferences to what extent they wish to be involved.
- There is a lack of insight into how family involvement plays out in the actual intensive care practice.

**What this paper adds**

- Doctors show eight communicative behaviors to involve families in the decision-making process.
- Sequences of doctors’ behaviors either reflected consistent approaches—a shared approach or a physician-driven approach—or reflected vacillating between both approaches.
- Doctors more often displayed a physician-driven or a vacillating approach than a shared approach.
- To all communicative behaviors families most commonly responded passively by only providing listening signals or a short confirmation.

**Implications for practice, theory, or policy**

- Tailoring doctors’ communication to families’ preferences and needs does not seem to be integrated into actual practice.
- Educational initiatives should focus on improving doctors’ skills in tactfully exploring families’ decision-making preferences, thoroughly querying patients’ wishes and life story, and in mutually sharing values and treatment preferences.
- Peer-to-peer coaching and recurring mirror interviews with families can create more awareness.

## Introduction

In intensive care units, decisions about the continuation or discontinuation of life-sustaining treatment are made almost daily.^1,2^ These decisions are particularly challenging due to time constraints, the absence of pre-existing relationships with patients and their families, and ethical dilemmas.^3^ These dilemmas often concern decisions in the “gray zone” in which there is incomplete knowledge about the relative harms and benefits of the remaining options and no best option exists.^4^ It is commonly advocated that patients’ values and preferences should be leading, especially when making decisions in the gray zone.^3,5,6^ Yet, most intensive care patients are not able to communicate their wishes.^7,8^ In these cases, doctors have to rely on family members, acting as the patient’s surrogate decision-makers.^2,9,10^ Families vary in their preference to what extent they wish to be involved in the decision-making process.^1,11–13^ Therefore, in each conversation, doctors have to find the right balance between sharing decisions with families on the one hand while protecting them from a responsibility they experience as too burdensome on the other hand.^14^

Recent studies conclude that finding this balance in involving families requires a tailored approach—in which families are involved according to their preferences—instead of a model based approach—in which one particular conversation model is followed without individualization.^3,15,16^ Tailoring may well enhance family satisfaction and reduce families’ later uncertainty, regret, or blaming the medical team for undesired outcomes.^17–19^ Families’ preferences and needs should therefore be leading, not the preferences of individual doctors nor the hospital’s advocated approach.^17,18,19^

Research on how family involvement plays out in actual practice is scarce. Previous retrospective studies indicate that important communicative opportunities are often missed, especially, listening and responding to families, acknowledging and addressing their emotions, and eliciting and incorporating their values and preferences.^8,10,20–27^

Our study aims to answer the research question *how* doctors actually involve families in the decision-making process regarding life-sustaining treatment on the neonatal, pediatric, and adult intensive care. We focus in particular on decisions in the gray zone.

## Methods

### Research question

How do doctors involve families in the decision-making process regarding life-sustaining treatment on the neonatal, pediatric, and adult intensive care?

### Design and setting

Data were derived from audio recordings of family conferences (henceforth: conversations) in the neonatal, pediatric, or adult intensive care of the Amsterdam UMC. “Families” refers to the family members or close friends who were present during these conversations.

The explorative analysis focused on doctors’ communicative behaviors to (not) involve families of patients in the decision-making process, using inductive thematic analysis. This technique seeks to identify and analyze themes and patterns in a qualitative data set.^28^ An inductive approach was chosen to explore patterns within the data.^29,30^

### Population

Families of 36 patients and 71 doctors participated. Table 1 lists their characteristics.

**Table 1.** Main characteristics of included patients, family members, and doctors.

Characteristics	Patients (*N* = 36), *n* (%)	Family members (*N* = 104), *n* (%)	Doctors (*N* = 71), *n* (%)
Setting			
Neonatal intensive care unit	12 (33)	33 (32)	22 (31)
Pediatric intensive care unit	12 (33)	30 (29)	35 (49)
Adult intensive care unit	12 (33)	41 (39)	14 (20)
Age (*y*)			
Premature	11 (30)	–	–
0–1	6 (16)	–	–
1–4	1 (3)	–	–
4–12	2 (6)	–	–
12–16	2 (6)	–	–
16–21	2 (6)	–	–
21–35	–	–	–
35–50	3 (8)	–	–
50–65	5 (14)	–	–
65+	4 (11)	–	–
Gender			
Male	17 (47)	41 (39)	28 (40)
Female	19 (53)	63 (61)	43 (60)
Main diagnosis			
Prematurity	5 (14)	–	–
Prematurity + congenital disorder + acute illness	1 (3)	–	–
Perinatal asphyxia	4 (11)	–	–
Congenital disorder	13 (36)	–	–
Acute illness	11 (30)	–	–
Cancer + acute illness	2 (6)	–	–
Neurological damage			
Yes	24 (67)	–	–
No	12 (33)	–	–
Total duration of care in the intensive care unit			
0–24 h	5 (14)	–	–
1–7 days	10 (28)	–	–
1–4 week	16 (44)	–	–
1–3 months	5 (14)	–	–
Relation to the patient			
Parent	–	46 (44)	–
Grandparent	–	8 (7)	–
Partner	–	7 (7)	–
Child	–	9 (9)	–
Sibling	–	8 (7)	–
Brother in law/Sister in law	–	2 (2)	–
Aunt/Uncle/Cousin	–	10 (10)	–
Friend	–	4 (4)	–
Other	–	5 (5)	–
Unknown	–	5 (5)	–
Medical specialty			
Neonatologist	–	–	14 (20)
Pediatric intensivist	–	–	9 (13)
Pediatrician	–	–	15 (21)
Pediatric neurologist	–	–	7 (10)
Pediatric cardiologist	–	–	3 (4)
Metabolic pediatrician	–	–	2 (3)
Pediatric pulmonologist	–	–	1 (1)
Intensivist	–	–	9 (13)
Anesthesiologist	–	–	4 (6)
Internist-hematologist	–	–	1 (1)
Neurosurgeon	–	–	3 (4)
Neurologist	–	–	1 (1)
Unknown	–	–	2 (3)
Role			
Resident	–	–	20 (28)
Fellow	–	–	13 (18)
Staff	–	–	36 (51)
Unknown	–	–	2 (3)

### Sampling

We used purposive sampling to reach a population with diversity in ethnic background, gender, age, and disease and by doing so to obtain diversity of perspectives.

### Recruitment

Previous to the data collection, all doctors, and nurses from the participating units received oral and written study information and were asked for their consent to participate. All doctors and all but one nurse (from the neonatal intensive care) gave their consent to participate.

The inclusion period lasted from April 2018 to December 2019. Families of patients were eligible to participate from the moment when doubts were expressed by the medical team and/or the family whether continuing life-sustaining treatment was in the patient’s best interest in light of their remaining quality of life.

### Data collection

The attending doctor or nurse introduced the study to eligible families. Interested families were further informed and asked for their oral and written consent by a member of the research team or the attending doctor.

From the moment of inclusion, all conversations (*n* = 101) between the medical team and families were audio recorded until a final decision was reached to either continue or discontinue life-sustaining treatment.

### Data analysis

**Figure 1.**
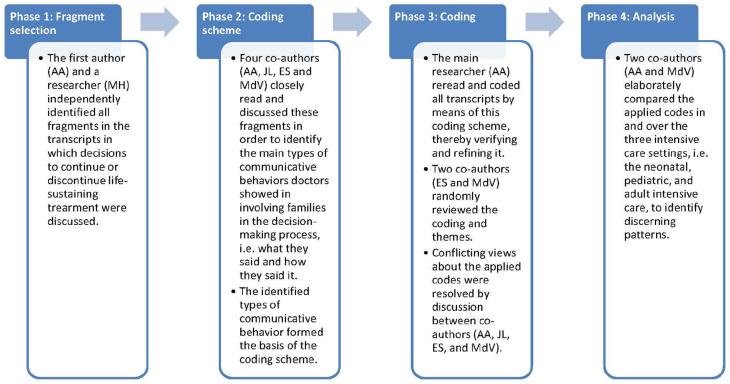
Four phases of coding and analysis.

The audio recordings were transcribed verbatim and anonymized. Transcripts (*n* = 101) were then uploaded to MaxQDA. We coded and analyzed our data by means of inductive thematic analysis.^28^ Coding and analysis consisted of four phases, as illustrated in Figure 1.

A process of reflection and discussion between co-authors (AA, JL, ES, and MdV) was used to identify and minimize bias. Efforts were made to ensure confirmability of the findings, through a review of the coding and themes by two co-authors (ES and MdV).

### Ethical considerations

The Amsterdam UMC institutional review board waived approval of this study (W17_475 # 17.548). Informed consent was acquired from one representative on behalf of the whole family. Families could withdraw their consent at any time.

## Results

We identified eight types of communicative behavior by which doctors involved families in decisions to continue or discontinue life-sustaining treatment. We found that these behaviors often occurred sequentially.

### Main types of communicative behavior

In Table 2, we exemplify doctors’ main types of behaviors with illustrative quotes and present the frequency of occurrence of each behavior.

**Table 2.** Main types of doctors’ communicative behavior in involving families in decisions to continue or discontinue life-sustaining treatment with illustrative quotes and frequencies per setting and overall.

Communicative behavior	Illustrative quote	Frequency of occurrence			
		Within neonatal intensive care (*N* = 55), *n* (%)	Within pediatric intensive care (*N* = 53), *n* (%)	Within adult intensive care (*N* = 69), *n* (%)	Overall (*N* = 117), *n* (%)
#1. Stressing that the medical team needs the family’s input and advice	“And that’s the reason why we talk with you. First, so that we can share with you what we do and do not know. Second, because we need your advice or, at least, your opinion.”	3 (5)	1 (2)	8 (12)	12 (7)
#2. Pointing out doctors’ preference or obligation to make the decision together with families	“This is never a decision which is made by one doctor. It is a decision made by the team. But we also make the decision together with you.”	6 (11)	10 (19)	4 (6)	20 (11)
#3. Querying the patient’s wishes and life story	“It is difficult to make a decision for someone who cannot answer our questions. So that’s why we would like to discuss your mother’s way of life with you.”	0 (0)	0 (0)	6 (9)	6 (3)
#4. Explicitly asking families to share their opinion regarding the decision at stake	“I have read the report which states that you do not want us to resuscitate her because of bad experiences acquaintances of yours have had with resuscitation. I think it is important to discuss this. Can you explain this a bit more? Because we can then better understand where you are coming from.”	7 (13)	7 (13)	1 (1)	15 (8)
#5. Explicitly asking for the family’s consent	“Do you agree with that?”	3 (5)	1 (2)	1 (1)	5 (3)
#6. Proposing a decision to continue or discontinue life-sustaining treatment	“My proposal is – and I will also discuss this with our group of intensivists tomorrow – to wait until Friday to see if she improves with this therapy.”	13 (24)	10 (19)	7 (10)	30 (17)
#7. Announcing a decision which will be or has already been made by the medical team	“So this implies that we as a medical team have decided to stop the medically futile treatment we are now providing.”	22 (40)	23 (43)	32 (46)	77 (44)
#8. Pointing out that making the decision is a medical responsibility	“The decision to continue or withdraw treatment is not yours to make. It is a decision made by the medical team.”	1 (2)	1 (2)	10 (15)	12 (7)

#### #1. Stressing that the medical team needs the family’s input and advice

“What would be appropriate and how long can we wait till we have to make a decision? We very much need you in that regard. Of course, we have our ideas about quality of life and what would be acceptable, but naturally that is a very gray zone. Because what we think may not be the same as what you think in this respect. So we need your advice as parents on this matter.”

This behavior was more common in the adult intensive care than in the neonatal and pediatric intensive care. In addition to this behavior, most doctors underlined that needing the family’s input did not imply that they assigned full decision-making responsibility to families. Often, they also explained that the quality of the patient’s life was an important element in making the right decision. For this reason, the families’ insights about the patient’s wishes and life story were needed to complement the knowledge of the medical team.

#### #2. Pointing out doctors’ preference or obligation to make the decision together with families

“We make the decision as a team. You don’t have to do that on your own. We decide as a team and therefore we also fully support it as a team. And when I say ‘team’, I also see you as part of this team.”

This behavior often co-occurred with communicative behavior #1, but differed in focus: behavior #1 revolved around the family’s input and advice as an essential component of decision-making, whereas behavior #2 focused on the decision-making itself. Contrary to doctors in the neonatal and pediatric intensive care, doctors in the adult intensive care did not generally show this behavior.

#### #3. Querying the patient’s wishes and life story

“And we would very much like to hear from you: who is your mother? Who is your wife? What suits . . . in this situation, what suits her best to do?”

This behavior overlapped with communicative behavior #1 but differed from it in the respect that in behavior #3 specific aspects regarding the patient’s wishes and life story are queried, whereas in behavior #1 the necessity of the family’s input and advice is stressed without necessarily asking for specific input or advice. This behavior was only seen in the adult intensive care, but not on a regular basis. Doctors queried the patient’s wishes by asking the family what the patient would have said if they would have been able to. In exploring the patient’s life story, doctors often used expressions like: “Tell me, what kind of person was your wife? And in that respect, what would suit her?” Doctors often added that the patient’s presumed wishes and life story formed an important component of the decision-making.

#### #4. Explicitly asking families to share their opinion regarding the decision at stake

“I want to know what you think. Where you stand on this issue. How you look at the future of your baby. If you say: ‘I definitely don’t want this’, then we will talk about it. Look, we won’t do anything you do not support.”

Doctors in the adult intensive care almost never explicitly asked families to share their opinion. This contrasted with their announcement that they would need the family’s input and advice (#1). Doctors in the neonatal and pediatric intensive care more often explicitly asked families to express their opinion, albeit in a minority of their conversations.

#### #5. Explicitly asking for the family’s consent

“Is this something you can support? . . . Is it something you can agree with?”

In all intensive care settings, doctors rarely asked for the family’s consent.

#### #6. Proposing a decision to continue or discontinue life-sustaining treatment

“For us this is a very important fact, knowing that from a medical perspective we cannot offer him anything else anymore and that his prospects are so bad that we as medical team, eh, actually intend to propose to stop treatment.”

This behavior was shown on a regular basis in all intensive care settings, but it was more common in the neonatal and pediatric intensive care than in the adult intensive care. In addition to proposing a decision, doctors often elaborately justified this decision by providing extensive prognostic information, by referring to the patient’s wishes and life story, or by making a moral appeal, for example: “We should give him a fair chance.” Moreover, they frequently stressed that the decision was the medically preferred one. In this respect, doctors frequently used the term “medical team”, thereby showing that the proposed decision was not their individual decision.

#### #7. Announcing a decision which will be or has already been made by the medical team

“The moment she would need CPR, so when her heart shows that it is simply too much and says: ‘I am too ill, this is not possible anymore’, then we will say: ‘enough is enough’. Then we will not actively resuscitate her. Ehm, based on medical grounds, we think that that’s a bridge too far.”

Whereas proposing (#6) is more of a suggestion with a request for an agreement, the announcement (#7) does not allow space for family involvement. In all intensive care settings, behavior #7 was the most frequently observed behavior. The necessity of the announced decision was generally presented as a logical consequence of the actual situation. Announcing a decision commonly took the form of a command: “We should . . .” or “You should . . .,” suggesting a sense of obviousness, thereby limiting the room for questioning. Often, doctors provided further explanation of or medical justifications for the decision. They sometimes offered room for families’ input regarding the implementation of the decision, for example: “Together we can decide how and when we’re going to withdraw treatment, but it’s certain that we will.”

#### #8. Pointing out that making the decision is a medical responsibility

“This decision to stop treatment . . . to withdraw, ehm, is based on medical grounds and is also supported by the medical team. Not by one doctor alone, but by the entire team. And I also want to explicitly unburden you in this respect.”

Contrary to doctors in the neonatal and pediatric intensive care, doctors in the adult intensive care regularly showed this behavior. They underlined that the decision was based on medical grounds or that it was up to the medical team rather than the family to make this decision. Sometimes, doctors stressed that family consent was unnecessary and/or would not be requested. In addition, they explained that bearing decision-making responsibility would be too burdensome for families.

**Figure 2.**
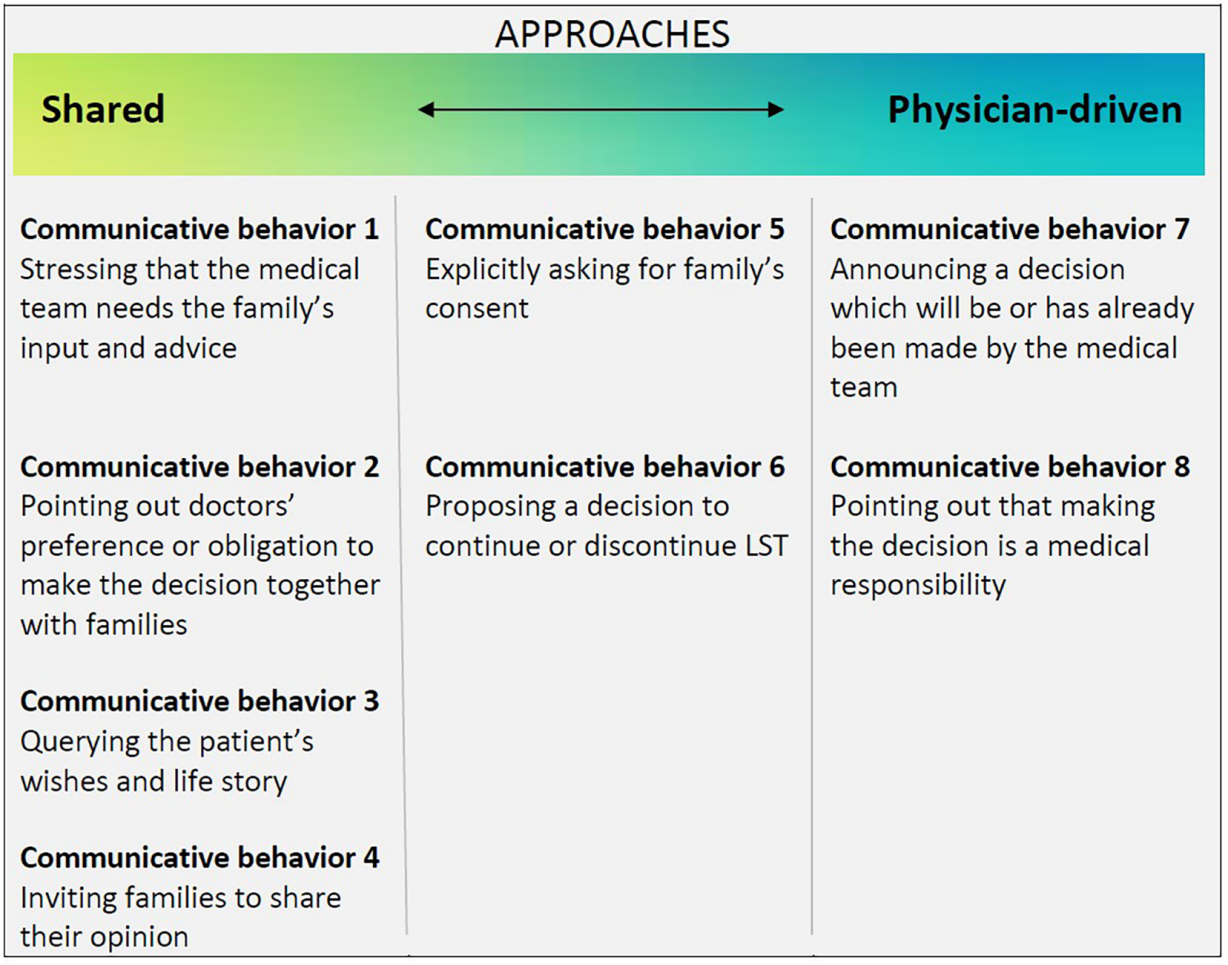
Overview of which behaviors reflected a shared approach, which behaviors reflected a physician-driven approach, and two in-between behaviors.

These eight communicative behaviors mainly reflected two key types of approaches: a shared approach and a physician-driven approach. As Figure 2 shows, most behaviors to a greater or lesser extent reflected either of these approaches, whereas behaviors #5 and #6 could be seen as a middle way. We did not find a relation between behavior or approach and withdrawing or continuing life-sustaining treatment.

Families responded in three main ways to all eight communicative behaviors. The first and most common type of response was *responding passively* by only providing listening signals (e.g. “Mm mm”) or a short confirmation such as “Yes, exactly.” In these instances, it remained unclear if families understood what they had been told and how they valued the doctor’s communicative behavior. Second, *families gave an active response that matched the doctors’ communicative behavior.* These responses were in line with the doctor’s question or remark (e.g. sharing input and advice when asked to). These first two types of responses were observed around all types of doctors’ communicative behavior. Third, *families gave an active response that did not match the doctor’s communicative behavior*. For instance, families occasionally casted doubt on the decision being presented to them (#6–7), for example: “I don’t know whether a cannula will be beneficial for him, but maybe you can convince me.” Incidentally, families stated that they would rather leave the decision-making to the doctors in response to an invitation to share their opinion (#4), for example: “I feel like I should put my trust in your expertise because you are better at estimating chances.”

### Sequences of doctors’ communicative behaviors

We commonly observed doctors referring to family involvement multiple times and in multiple ways. Their sequential communicative behaviors either reflected consistent approaches—a shared approach or a physician-driven approach—or reflected vacillating between both approaches. In a vast amount of conversations in all three intensive care settings, we observed sequences reflecting a physician-driven approach (#7–8, occasionally in combination with the “in-between” communicative behaviors #5–6). This approach mainly yielded passive and compliant responses from families. Occasionally, family members responded in a more active way, for instance by showing their disagreement or by actively sharing their views even though they were not invited to do so.

We observed sequences reflecting a shared approach (#1–4, occasionally in combination with the “in-between” communicative behaviors #5–6) in a minority of the conversations from the neonatal and pediatric intensive care and in none of the conversations from the adult intensive care. When doctors showed a shared approach, families often, albeit not always, responded in an explicit, and active manner. Families, for instance, shared their views, discussed the patient’s wishes or gave their consent.

**Figure 3.**
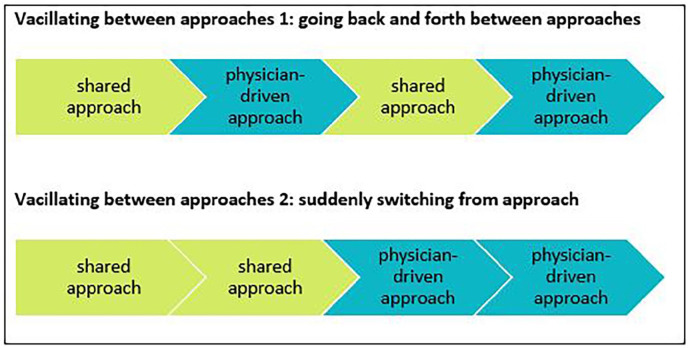
Simplified visualization of the two ways in which doctors vacillated between approaches.

Additionally, we identified sequences which reflected both types of approaches. Within these sequences, doctors vacillated between a shared approach and a physician-driven approach. They did so in two ways, as visualized in Figure 3. In the first way, doctors kept moving back and forth between a shared approach and a physician-driven approach. In the second way, doctors quite suddenly switched from a shared approach to a physician-driven approach, somewhere in the middle of the conversation. We observed a vacillating approach to occur frequently in conversations in all three intensive care settings. We detected two main patterns. Most commonly, doctors presented or proposed a decision and afterwards explored whether this decision indeed fitted the patient and/or the families, regardless of families’ verbal behavior. Occasionally, doctors switched from a shared to a physician-driven approaches in response to families’ verbalized worries about their decision-making responsibility. In both patterns, doctors did not necessarily continue the approach which they switched to, but also commonly switched back to their original approach. Families did not show apparent confusion in response to these seemingly contradictory behaviors.

In none of the conversations, families were invited to make explicit how they wished to be involved. Occasionally, they explained this preference spontaneously, with some families stating that they should be the final decision-maker while others stated that they wished to leave the decision-making responsibility to the doctor. In conclusion, doctors’ communicative behaviors were generally not based on families’ explicitly verbalized communication preferences and needs.

## Discussion

We aimed to explore *how* doctors actually involve families in decisions concerning the continuation or discontinuation of life-sustaining treatment. Our results show that in a majority of conversations, doctors displayed a variety of communicative behaviors to involve families in the decision-making process.

Not all identified behaviors and sequences were equally common. Most common was the communicative behavior in which doctors just informed families about the decision (to be) made (#7). Conversely, doctors rarely queried families about the patient’s wishes and life story (#3) and explicitly asked for families’ consent (#5). This can be seen as a missed opportunity, because both behaviors enable families to share their views. This in turn will help to make the most appropriate decision for the patient.^31^ Moreover, it will help families in their acceptance and coping.^6,13,20,32^ The same applies to querying families’ own views on the situation and on the decision at stake (#4). We only observed this communicative behavior in the neonatal and pediatric intensive care, but rarely.

In line with the outcomes of previous retrospective studies, we observed that doctors more often displayed a physician-driven or a vacillating approach than an unambiguous shared approach.^7,22,25^ This may be explained by the fact that the decisions at stake concerned crucial decisions about life and death. Doctors may consider a shared approach not suitable and appropriate for this specific type of decisions. This may (partly) be the result of their conviction that families should be protected from the burden of responsibility and potential feelings of guilt.^7,11,16,20,33–35^

Vacillating between a shared and a physician-driven approach can be seen as a (unconscious) strategy by doctors to cope with their “double ethical duty” of finding the right balance between involving families and protecting them from too much responsibility. In this respect, it can also be seen as a way of tailoring communication in which seemingly contradictory behaviors are, in fact, complementary. However, we found no examples of doctors verifying whether their approaches matched the families’ decision-making preferences. Doctors only explicitly discussed their own perspective (#1–2, #8); they did not ask families about their perspective on this issue.

As former studies have underlined, families vary in their preference to what extent they wish to be involved.^2,5,11–13,24,36^ As a result, international guidelines recommend that doctors should tailor their communication to the preferences and needs of individual families.^5,15,16^ This may well enhance family satisfaction and reduce their later uncertainty, regret and blaming the medical team for undesired outcomes.^17–19^

The doctors in our study did not offer a lot of room for family input. This corresponds with the outcomes of previous studies.^8,20–22,25^ Yet, when doctors invited families to share their views, most families then seized this opportunity. However, families incidentally replied that they rather left the decision-making to the doctors when they were asked to share their opinion (#4). This may suggest that #4 is not the most effective way to elicit family input and in fact querying the patient’s wishes and life story (#3) might be a more fruitful approach. This is in line with Shaw et al.’s^37^ finding that doctors’ communicative behaviors seem to influence to which extent families participate actively. All in all, this seems to be an indication for the existing power asymmetry between doctors and families and for doctors’ significant influence on family involvement.^7,14,22,27,38^

Our study indicates differences in doctors’ communicative behaviors between the neonatal and pediatric intensive care on the one hand, and the adult intensive care on the other. This may be explained by the fact that these settings fundamentally differ in several respects. First, due to the higher average age of patients, doctors and families in the adult intensive care may be more likely to acquiesce in the death of a patient. Moreover, as patients in this setting generally have had a longer life as compared to patients in the neonatal and pediatric intensive care, consequences of decision-making in the neonatal and pediatric intensive care may be perceived as carrying more weight—also ethically—and as requiring family involvement.^39^ Second, the relation between surrogate decision-maker and patient differs between intensive care settings. In the neonatal and pediatric intensive care, the surrogate decision-makers usually are the parents. In the adult intensive care, the surrogate decision-makers often are the partners, children, or siblings of the patient. The responsibility for making decisions for their child is more self-evident for parents (who also have formal responsibility for their child in other situations) than it is for families of patients who would normally be capable to decide for themselves.^39,40^ Recent studies have underlined that most parents of critically ill children prefer a shared approach, including making the final decision together with their child’s doctor.^18,21,26,41,42^ Third, the total duration of care generally was longer in the neonatal and pediatric intensive care than in the adult intensive care. This provides more time to build a trusting relationship between the medical team and the patient’s family, which may result in a more shared process of decision-making.^8,21,32,40^ Fourth, in all cases, at the point of inclusion all treatment decisions concerned decisions in the gray zone. However, in contrast to the neonatal and pediatric intensive care, decisions in the adult intensive care may more rapidly turn from “gray” into “black” because these decisions mainly concern older fragile patients, often with multiple comorbidities. The Dutch Medical Treatment Act states that if treatment has become futile, family’s input is not mandatory.^43^ However, it still remains important to check whether families assent to the decision to discontinue life-sustaining treatment, as this may lessen their later feelings of doubt, regret, and guilt.^17,44,45^

As are the Netherlands, other European countries and Asian, Middle-Eastern, and South-American countries are characterized by a more paternalistic medical and public opinion on making end-of-life decisions.^34,46,47–54^ By contrast, families of incapacitated patients are considered the primary decision-makers in the United States and Canada, based on prevailing moral and legal traditions.^53,55,56^ Despite these cultural differences, countries experience similar dilemmas of whether, in how far, and how to involve families of patients in the decision-making process.^39^ Our exploration can thus be regarded internationally relevant.

### Limitations and strengths

We have pushed for maximum variation regarding the participating doctors and families, selection bias may nevertheless have occurred. Another limitation is that we did only audio record conversations to minimize the intrusiveness of our data collection, which precluded the analysis of non-verbal communication. Moreover, we cannot rule out that the Hawthorne effect may have occurred. A third limitation is that the analysis focused mostly on the communicative behaviors of doctors, without considering how families’ communicative behaviors may have impacted this. It would be interesting if future research focused on this different perspective. Last, this study describes the practices within one medical center only, which was unavoidable given the logistical demands of the study.

The main strength of this study is that we investigated what actually happened in the decision-making conversations rather than what participants in retrospect thought had happened. Furthermore, we used a data-driven coding scheme. This resulted in an extensive overview of doctors’ communicative behaviors to involve families in critical and crucial decisions. As such, this study provides a comprehensive basis for future qualitative and quantitative multicenter research.

## Interpretation

The main implication of this study is that even though tailoring doctors’ communication to the families’ preferences and needs is advocated, it does not seem to be integrated into actual practice in the neonatal, pediatric, and adult intensive care. To allow for true tailoring, doctors’ awareness regarding their communicative behaviors is key. For this purpose, two simple questions can be helpful: (1) “Which communicative behaviors do/did I use in this situation?” and (2) “Why do/did I use these behaviors?” Two important factors to consider in answering the latter question are: “how gray is the decision?” and “does my approach fit this family’s communication preferences and needs?”. In order to create more awareness, peer-to-peer coaching in which doctors can observe and discuss their practices in and over their intensive care settings is a helpful tool. The same holds true for organizing recurring mirror interviews with families about their experiences regarding their involvement in the decision-making process for their critically ill family member.

## Appendix

### Appendix 1: Data analysis procedure

The audio recordings were transcribed verbatim and anonymized. Transcripts (*n* = 101) were then uploaded to MaxQDA. We coded and analyzed our data by means of inductive thematic analysis^29^. Coding and analysis consisted of four phases:

1. The first author (AA) and a researcher (MH) independently identified all fragments in the transcripts in which decisions to continue or discontinue life-sustaining treatment were discussed.
2. Four co-authors (AA, JL, ES, and MdV) closely read and discussed these fragments in order to identify the main types of communicative behaviors doctors showed in involving families in the decision-making process, that is, what they said and how they said it.
3. The identified types of communicative behavior formed the basis of the coding scheme.
4. The main researcher (AA) reread and coded all transcripts by means of this coding scheme, thereby verifying and refining it.
5. Two co-authors (ES and MdV) randomly reviewed the coding and themes.
6. Conflicting views about the applied codes were resolved by discussion between co-authors (AA, JL, ES, and MdV).
7. Two co-authors (AA and MdV) elaborately compared the applied codes in and over the three intensive care settings, that is, the neonatal, pediatric, and adult intensive care, to identify discerning patterns.

A process of reflection and discussion between co-authors (AA, JL, ES, and MdV) was used to identify and minimize bias. Efforts were made to ensure confirmability of the findings, through a review of the coding and themes by two co-authors (ES and MdV).
